# Drug-induced colitis on BRAF and MEK inhibitors for BRAF V600E-mutated non-small cell lung cancer: a case report

**DOI:** 10.1007/s10637-021-01166-7

**Published:** 2021-08-26

**Authors:** Francesco Gelsomino, Alessandro Di Federico, Maria Lucia Tardio, Giada Grilli, Antonietta D’Errico, Andrea Ardizzoni, Stefania Salvagni

**Affiliations:** 1 Medical Oncology Unit, IRCCS Azienda Ospedaliero-Universitaria Di Bologna, Bologna, Italy; 2 Department of Specialized, Experimental and Diagnostic Medicine, University of Bologna, Bologna, Italy; 3 Anatomy and Histopathology Unit, IRCCS Azienda Ospedaliero-Universitaria Di Bologna, Bologna, Italy

**Keywords:** Non-small cell lung cancer, BRAF, MEK, Colitis, Gastrointestinal toxicity

## Abstract

*
Introduction.* The combination of BRAF and MEK inhibitors has deeply changed the treatment of *BRAF V600-*mutant non-small cell lung cancer patients. These agents demonstrated high antitumor activity as well as safe and manageable toxicity profile. Hypertension, pyrexia and increased liver enzymes are the most common adverse events. Gastrointestinal toxicities are rare, and mainly consist of mild grade vomiting and diarrhea. *Case report*. We report the case of 70-year-old man affected by *BRAF V600*-mutant NSCLC with bilateral lung and bone metastases. First-line treatment with encorafenib (450 mg once daily) and binimetinib (45 mg twice daily) was administered within a clinical trial. At the first radiological assessment, computed tomography (CT) scan showed a partial response and signs of intestinal inflammation were reported. The investigational treatment was timely withheld. The subsequent colonoscopy demonstrated the presence of ulcerative lesions at the caecal tract, and the histological diagnosis suggested a drug-induced colitis. No specific treatment was given as the patient did not report abdominal disturbances. Forty-five days after treatment interruption a new CT scan showed the resolution of bowel inflammation and investigational treatment was resumed at the same doses. The patient is still alive and free of toxicity recurrence after 11 months from treatment initiation. *Conclusion*. Severe gastrointestinal toxicities are uncommon with BRAF and MEK inhibitors, although cases of colitis and intestinal perforation have already been reported in literature. The pathogenesis seems to be related to the MAPK pathway inhibition performed by MEK inhibitors. These adverse events should be accounted given the potential to evolve into life-threatening conditions.

## Introduction

*BRAF* gene mutations can be detected in approximately 2–4% of advanced non-small cell lung cancer (NSCLC) patients. About half of them are *V600E* mutations that determine the constitutive activation of the *BRAF* kinase domain, leading to cancer growth, proliferation and survival [1, 2]. Recently, the evidence of high antitumor activity as well as a safe and manageable toxicity profile of dabrafenib, a BRAF inhibitor, and trametinib, a MEK inhibitor, allowed this combination to become a new standard-of-care for *BRAF V600*-mutant NSCLC patients [3, 4]. Novel combinations of BRAF and MEK inhibitors, such as encorafenib and binimetinib, are under evaluation (NCT03915951). Liver function tests and creatine phosphokinase increase, hypertension and pyrexia are the most frequently reported grade ≥ 3 adverse events. Severe gastrointestinal toxicities, mostly abdominal pain, diarrhea and vomiting, had low incidence in the clinical trials testing these agents [3, 4].

## Case report

**Fig. 1:**
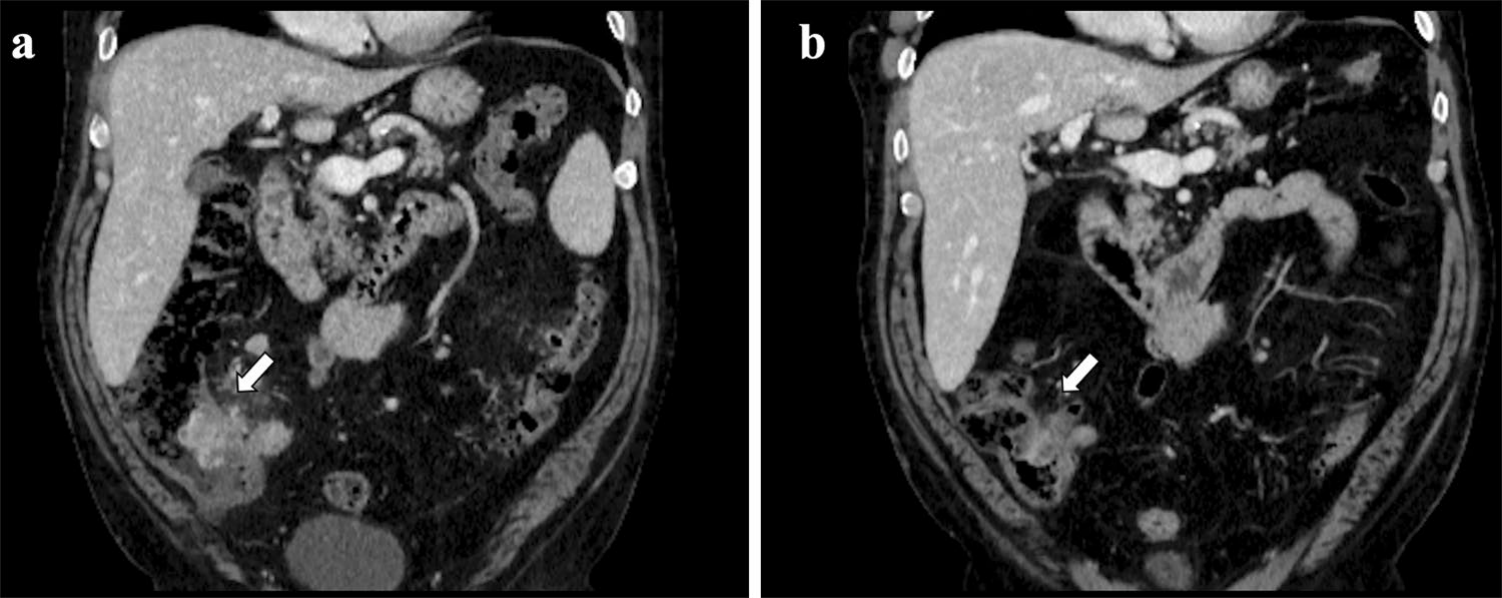
A 7-cm long contrast-enhanced marked thickening of the last ileal tract, along with the involvement of caecum and appendix, perivisceral adipose tissue suffusion and multiple enlarged lymph nodes at CT scan carried out 2 months after the beginning of BRAF/MEK TKIs (**a**); almost complete remission of previous radiological findings at CT scan performed after 1 month from treatment interruption (**b**)

**Fig. 2:**
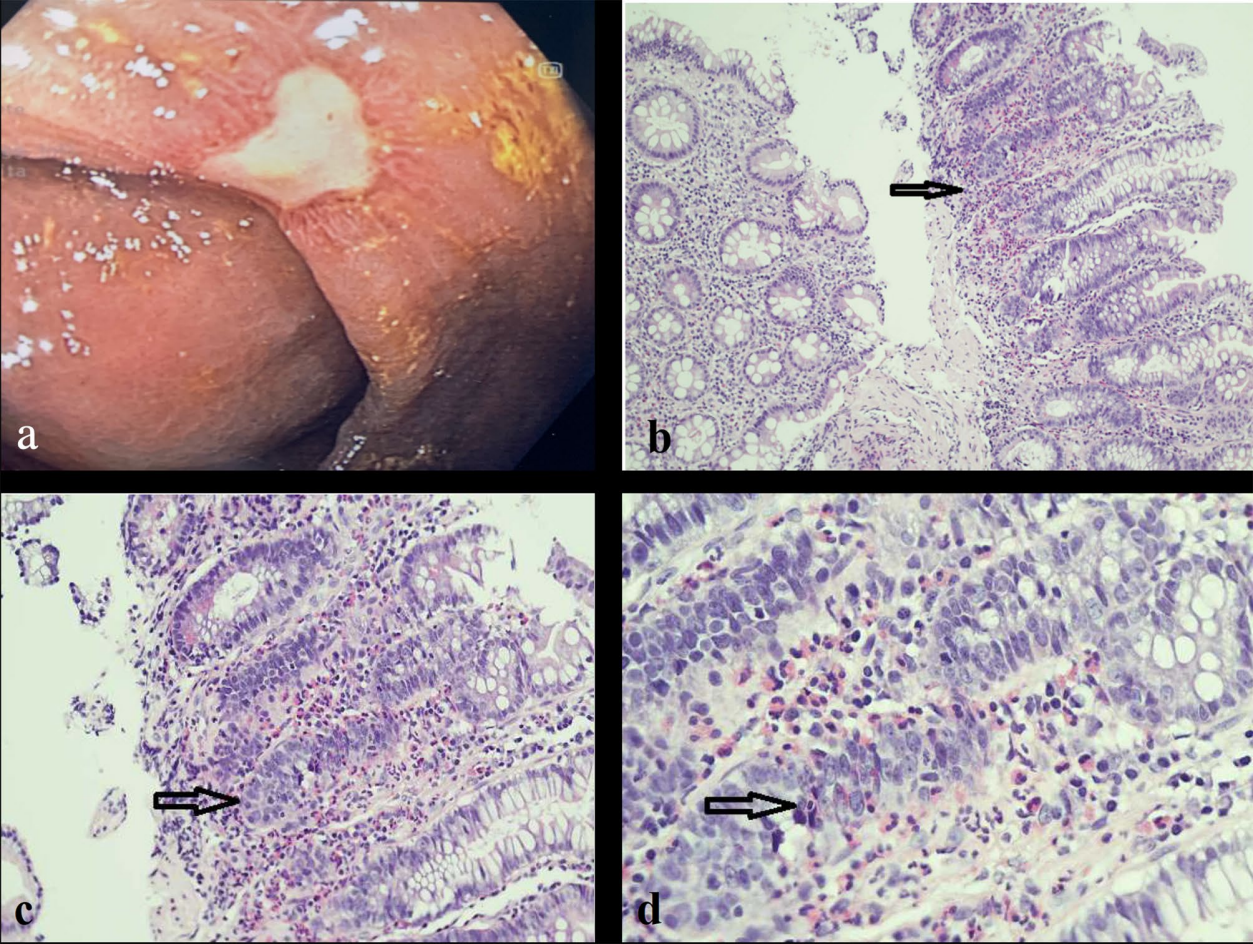
Endoscopic imaging showing the largest ulcerative lesion (1.5 cm) of the caecum with fibrinous and granulation tissue, as per reparative processes (**a**); corresponding microscopic examination at 10x (**b**), 20x (**c**) and 40x (**d**) magnification. The glands are normally oriented, with a marked chronic inflammatory infiltrate and eosinophils (more than 60/40x, see arrow). Note the increased eosinophils (more than 60/40x) both in the lamina propria and within the glands (arrow). These features are associated with drug-induced mucosal eosinophilia

Herein, we report our experience with a 70-year-old man diagnosed with a *BRAF*^*V600E*^-mutant, PD-L1 positive (tumor proportion score 90%) adenocarcinoma of the lung. Baseline CT scan showed bilateral lung lesions and bone dissemination. The patient received the combination of encorafenib (450 mg once daily) and binimetinib (45 mg twice daily) as upfront treatment within a clinical trial (NCT03915951). Two months after starting treatment the radiological assessment showed a partial response (45% decrease of target lesions per RECIST 1.1). As incidental finding, a contrast-enhanced increased thickness of the last ileal loop associated with perivisceral fat suffusion and enlarged lymph nodes was detected (Fig. 1a). Baseline CT scans were reviewed and no inflammatory finding was detected at that level. The treatment was withheld in suspected bowel inflammatory disease and a colonoscopy was performed. The intestinal endoscopy showed a diffuse mucosal erythema of the right upper colon and the presence of two ulcerative lesions at the caecal tract (Fig. 2a). The pathologic examination showed a mixed inflammatory infiltrate in the lamina propria of the caecum, associated with severe eosinophilia. Neither intraepithelial lymphocytes nor epithelial apoptotic bodies, known to be related to immune-mediated damage, were detected (Fig. 2b-d) and CD4/CD8 ratio was more than 1. All these findings suggested a diagnosis of drug-induced colitis.

No specific treatment was given as the patient did not report abdominal disturbances. Based on the evidence of complete recovery of prior radiological findings at a CT scan (Fig. 1b) performed 45 days after treatment interruption, the investigational drugs were resumed at the same doses. The patient is still alive 11 months after the start of treatment and he continues to take encorafenib and binimetinib at full doses with no evidence of toxicity recurrence.

## Discussion

Diarrhea and vomiting were the most frequent grade ≥ 3 gastrointestinal toxicities in the clinical trials testing BRAF inhibitors alone or combined with MEK inhibitors [3–5]. Rare cases of colitis and intestinal perforation have been reported [6–9]. The Ras-MEK-ERK pathway plays a crucial role in the proliferation, differentiation, migration and survival of the gastrointestinal epithelium. The underlying mucosal damage that causes colitis might be related to the inhibition of this signalling pathway by MEK inhibitors [10].

These adverse events seem to have a higher incidence combining BRAF and MEK inhibitors than to BRAF inhibitors alone. A correct management of these treatment-related adverse events requires the treatment withdrawal, that will be resumed at reduced dose at the resolution of the gastrointestinal toxicity [11]. Mourad et al. [6] conducted a retrospective analysis of severe gastrointestinal toxicities in melanoma patients treated with BRAF and MEK inhibitors alone or combined. They described three cases of colitis (2 of them treated with BRAF and MEK inhibitors and 1 treated with MEK inhibitor alone), all of them presenting as watery diarrhea. Colitis resolved following treatment withdrawal, and contrary to the management of our patient the MEK inhibitor was not resumed. Moreover, two patients treated with BRAF and MEK inhibitors developed intestinal perforation that required urgent surgical management, leading to a permanent ileostomy in one case. Other cases of intestinal perforation associated to MEK inhibitors have been described, although in some cases tumor regression in response to treatment may be the underlying cause of the event itself [7–9].

Notably, we initially assumed that our case was an immune-mediated colitis. However, following an accurate histological examination including immune cell staining and CD4/CD8 ratio, it was excluded as the main pathological mechanism. In fact, immune related adverse events (irAEs), which have been typically described in patients treated with immune-checkpoint inhibitors, are an emerging type of toxicity associated with BRAF and MEK inhibitors [12]. Ben-Betzalel and colleagues described possible irAEs developing in 10 patients on BRAF ± MEK inhibitors. The immune-mediated mechanism was supposed due to the nature of the event (vitiligo, uveitis, erythema nodosum and keratitis sicca), as confirmatory biopsies were not performed [12]. Patients who developed possible irAEs showed higher response rate, deeper tumor responses and prolonged progression-free survival. In light of this finding, it is important to accurately characterize suspected irAEs given the prognostic role that these may retain.

In conclusion, the risk of developing colitis should be accounted in patients treated with BRAF and MEK inhibitors, as it represents an uncommon adverse event with the potential to evolve into life-threating conditions, such as intestinal perforation. A timely and accurate histopathological characterization of the lesion might provide relevant prognostic and therapeutic implications.

## Data Availability

The current research was entirely conducted in our Institution.
